# Predictive value of HTS grade in patients with intrahepatic cholangiocarcinoma undergoing radical resection: a multicenter study from China

**DOI:** 10.1186/s12957-023-03281-6

**Published:** 2024-01-11

**Authors:** Guan Huang, Haofeng Zhang, Zhenwei Yang, Qingshan Li, Hao Yuan, Pengyu Chen, Chenxi Xie, Bo Meng, Xianzhou Zhang, Kunlun Chen, Haibo Yu

**Affiliations:** 1 Department of Hepatobiliary Surgery, People’s Hospital of Zhengzhou University, Zhengzhou, Henan Province China; 2 Department of Hepatobiliary Surgery, People’s Hospital of Henan University, Zhengzhou, Henan Province China; 3 Department of Hepatobiliary Surgery, Henan Province People’s Hospital, Zhengzhou, Henan Province China; 4 Department of Hepatobiliary Surgery, Affiliated Cancer Hospital of Zhengzhou University, Zhengzhou, Henan Province China; 5 Department of Hepatobiliary Surgery, The First Affiliated Hospital of Zhengzhou University, Zhengzhou, Henan Province China

**Keywords:** HTS grade, Intrahepatic cholangiocarcinoma, Radical surgery, Nomogram, Prognosis

## Abstract

**Background:**

Intrahepatic cholangiocarcinoma (ICC) is a highly malignant tumor with a poor prognosis. This study aimed to investigate whether Hemoglobin, Albumin, Lymphocytes, and Platelets (HALP) score and Tumor Burden Score (TBS) serves as independent influencing factors following radical resection in patients with ICC. Furthermore, we sought to evaluate the predictive capacity of the combined HALP and TBS grade, referred to as HTS grade, and to develop a prognostic prediction model.

**Methods:**

Clinical data for ICC patients who underwent radical resection were retrospectively analyzed. Univariate and multivariate Cox regression analyses were first used to find influencing factors of prognosis for ICC. Receiver operating characteristic (ROC) curves were then used to find the optimal cut-off values for HALP score and TBS and to compare the predictive ability of HALP, TBS, and HTS grade using the area under these curves (AUC). Nomogram prediction models were constructed and validated based on the results of the multivariate analysis.

**Results:**

Among 423 patients, 234 (55.3%) were male and 202 (47.8) were aged ≥ 60 years. The cut-off value of HALP was found to be 37.1 and for TBS to be 6.3. Our univariate results showed that HALP, TBS, and HTS grade were prognostic factors of ICC patients (all *P* < 0.05), and ROC results showed that HTS had the best predictive value. The Kaplan–Meier curve showed that the prognosis of ICC patients was worse with increasing HTS grade. Additionally, multivariate regression analysis showed that HTS grade, carbohydrate antigen 19–9 (CA19-9), tumor differentiation, and vascular invasion were independent influencing factors for Overall survival (OS) and that HTS grade, CA19-9, CEA, vascular invasion and lymph node invasion were independent influencing factors for recurrence-free survival (RFS) (all *P < *0.05). In the first, second, and third years of the training group, the AUCs for OS were 0.867, 0.902, and 0.881, and the AUCs for RFS were 0.849, 0.841, and 0.899, respectively. In the first, second, and third years of the validation group, the AUCs for OS were 0.727, 0.771, and 0.763, and the AUCs for RFS were 0.733, 0.746, and 0.801, respectively. Through the examination of calibration curves and using decision curve analysis (DCA), nomograms based on HTS grade showed excellent predictive performance.

**Conclusions:**

Our nomograms based on HTS grade had excellent predictive effects and may thus be able to help clinicians provide individualized clinical decision for ICC patients.

## Introduction

Intrahepatic cholangiocarcinoma (ICC) is the second most common primary hepatic malignant tumor after hepatocellular carcinoma and carries with it a poor prognosis as well as a rising worldwide incidence [1, 2]. Anatomically, ICC is a malignant tumor of the liver that originates from bile duct cells near the secondary bile ducts in the liver parenchyma [3]. Common risk factors for the occurrence of ICC are hepatolithiasis, cirrhosis, viral hepatitis, biliary cysts, and primary sclerosing cholangitis (PSC) [4]. At present, radical surgical resection remains the only effective treatment for ICC, but few patients are actually able to undergo surgical treatment [5]. What’s more, even after undergoing radical surgery, the 5-year survival rate is still only 20–40% [6, 7]. The prognosis of ICC patients is poor even with surgical resection due to the disease’s high recurrence rate, which is as high as 54–71% [8]. However, if clinicians can make use of a new prediction model to predict the prognosis of ICC patients in order to give each patient a personalized treatment plan, the devastation brought by ICC may be mitigated.

Systemic inflammatory response and nutritional status both have an important influence on the occurrence and development of malignant tumors [9, 10]. Recently, the score composed of hemoglobin, albumin, lymphocytes, and platelets (HALP) has been introduced to reflect systemic inflammation and nutritional status at the same time and has proven to be an effective predictor for the prognosis of ICC [11, 12]. Tumor Burden Score (TBS) is also a new index of ICC prognosis based on tumor size and tumor number. Sasaki et al. first proposed the use of TBS and applied it to patients with liver metastasis from colorectal cancer [13], and recent research shows that TBS has excellent clinical value in the prognosis of patients with hepatocellular carcinoma and ICC [14, 15]. Overall survival (OS) and recurrence-free survival (RFS) were our primary study endpoints.

In this study, we discuss the relationship between HALP and TBS and the prognosis of ICC, and put forward a new index, HTS, which combines the advantages of both HALP and TBS, and we analyze whether HTS had an advantage in predicting the prognosis of ICC compared to HALP and TBS. In addition, we also constructed predictive nomograms for ICC since nomograms are now widely used to predict the occurrence, development, and prognosis of various malignant tumors by comprehensively evaluating various risk factors [16, 17].

## Methods

### Study population

We retrospectively analyzed the clinical data of 423 patients with ICC who underwent radical resection in People's Hospital of Zhengzhou University, Affiliated Cancer Hospital of Zhengzhou University and The First affiliated hospital of Zhengzhou University between 2013 and 2021. Among them, there were 227 cases in People's Hospital of Zhengzhou University, 146 cases in Affiliated Cancer Hospital of Zhengzhou University hospital and 50 cases in The First affiliated hospital of Zhengzhou University hospital. The inclusion criteria were as follows: (1) The pathological results confirmed ICC; (2) Chemotherapy, radiotherapy and other auxiliary treatments were not given before operation; (3) No history of tumor in other systems; and (4) Complete clinical data. The exclusion criteria were: (1) The presence of preoperative extrahepatic metastases; (2) Pathological results showing that the margin was positive; (3) Suffering from other serious basic diseases (e.g. severe hypertension or diabetes, etc.) or (4) Complete absence of follow-up data. Patients from People's Hospital of Zhengzhou University hospital were used as the training cohort, and patients from the Affiliated Cancer Hospital of Zhengzhou University and The First affiliated hospital of Zhengzhou University were used as the validation cohort.

### HALP, TBS, and HTS Grade

The HALP score consists of hemoglobin, albumin, lymphocytes, and platelets and is calculated according to the formula HALP = hemoglobin (g/L) × albumin (g/L) × lymphocytes (10^9/L) / platelets (10^9/L). Patients were divided into high and low levels according to a HALP cut-off value (below). TBS is defined as the distance from the origin of the Cartesian plane spanned by the following two variables: maximum tumor diameter and number of tumors. The specific formula for calculating TBS is TBS^2^ = (maximum tumor diameter)^2^ + (number of tumors)^2^. Patients were also divided into high and low grades for TBS according to the cut-off value (below) obtained from ROC curve analysis. We constructed HTS grade by grouping according to HALP and TBS levels as follows: patients with high HALP/ low TBS level were classified as HTS grade 1, patients with high HALP/ high TBS level or low HALP/ low TBS level were classified as HTS grade 2, and patients with low HALP/ high TBS level were classified as HTS grade 3 (Table 1).

**Table 1:** Definition of HTS grade

HTS grade	Define conditions
1	HALP ≥ 37.1,TBS < 6.3
2	HALP < 37.1,TBS < 6.3 or HALP ≥ 37.1, TBS > 6.3
3	HALP < 37.1,TBS ≥ 6.3

*HALP* Hemoglobin, albumin, lymphocyte, and platelet, *TBS* Tumor burden score, *HTS* HALP and TBS

### Follow-Up

Follow-up was conducted by outpatient and inpatient review. Telephone follow-up surveys were conducted for patients who did not return to the hospital for re-examination. Patients were followed-up with every month for 6 months, then every 3 months for 2 years, and every 6 months after surgery until November, 2022. Examinations included: serum tumor markers, ultrasound, and enhanced CT. Overall survival (OS) is defined as the time from surgery until either death from any cause or until the last follow-up. RFS is the time from surgery to the earliest recurrence, or from surgery without recurrence to death due to ICC, or until the last follow-up.

### Statistical analysis

Continuous and categorical variables were reported as medians [interquartile range (IQR)] and frequencies (%), respectively. Continuous variables were compared using Student's *t*-test, the Wilcoxon rank sum test, or one-way ANOVA test, and categorical data were compared using *χ*^2^ tests or Fisher’s exact test. We used univariate and multivariate Cox proportional risk regressions to find independent influencers for OS and RFS and used ROC curves to compare their predictive values. Kaplan–Meier curves were also plotted to describe OS and RFS, and log-rank tests were used to examine the differences between them for various patient subgroups. Based on the results of our multivariate Cox proportional risk regression, we then used R software (version 4.3.0; R Foundation for Statistical Computing, Vienna, Austria) to create nomograms, and evaluation of the predictive performance of the nomograms using ROC AUC, calibration curves, and decision curve analysis (DCA). DCA is a method used to quantify the net benefit (NB) across various probability thresholds, allowing us to assess whether the advantages of a predictive model outweigh its limitations when guiding clinical decisions [18]. For all tests, *P*-values < 0.05 were considered to indicate statistically significant test results. SPSS version 25 (IBM Corp., Armonk, NY, USA) was employed for baseline data comparison, univariate and multivariate analysis. Kaplan–Meier and ROC curves were created using GraphPad Prism (version 9.0; San Diego, CA, USA). The R software utilized the following packages: "rms," "survival," "ggplot2," and "ggDCA."

## Results

### Patient population

We included 423 cases in total in this study. As summarized in Table 2, males accounted for 234 cases (55.3%), and 202 cases (47.8%) were aged ≥ 60 years. There were 153 cases (36.2%) with a history of hepatitis B infection, and the level of CA19-9 increased for 271 cases (64.1%). Tumor diameter ≥ 5 cm accounted for 282 cases (66.7%), and tumor multiplicity was present in 113 cases (26.7%). In the training group, the median survival time was 17 months and the median follow-up time was 15 months. In the validation group, the median survival time was 15 months and the median follow-up time was 12 months. Finally, the baseline data of the training group and the validation group were different in the following aspects: hepatolithiasis, Alanine Aminotransferase (ALT), alpha fetoprotein (AFP) and albumin level.

**Table 2:** Baseline characteristics of all ICC patients

Factors	Training group (*n* = 227)	Validation group (*n* = 196)	*t*/*χ*^*2*^ Value	*P*-Value
Age, year			1.112	0.292
< 60	124 (54.6)	97 (49.5)		
≥ 60	103 (45.4)	99 (50.5)		
Gender			0.255	0.614
Female	104 (45.8)	85 (43.4)		
Male	123 (54.2)	111 (56.6)		
History of hepatitis B infection			< 0.001	0.983
Negative	145 (63.9)	125 (63.8)		
Positive	82 (36.1)	71 (36.2)		
Hepatolithiasis			11.634	0.001
Negative	194 (85.5)	187 (95.4)		
Positive	33 (14.5)	9 (4.6)		
ALT (U/L)			4.755	0.029
< 50	173 (76.2)	166 (84.7)		
≥ 50	54 (23.8)	30 (15.3)		
AST (U/L)			2.104	0.147
< 40	162 (71.4)	152 (77.6)		
≥ 40	65 (28.6)	44 (22.4)		
Bilirubin (umol/L)			0.298	0.585
< 21	189 (83.8)	167 (85.2)		
≥ 21	38 (16.7)	29 (14.)		
AFP (ng/mL)			5.890	0.015
< 7	148 (65.2)	149 (76.0)		
≥ 7	79 (34.8)	47 (24.0)		
CEA (ng/mL)			2.303	0.129
< 5	155 (68.3)	120 (61.2)		
≥ 5	72 (31.7)	76 (38.8)		
CA19-9 (U/mL)			0.008	0.930
< 37	82 (36.1)	70 (35.7)		
≥ 37	145 (63.9)	126 (64.3)		
Tumor differentiation			0.607	0.436
Well-moderate	172 (75.8)	142 (72.4)		
Poor	55 (24.2)	54 (27.6)		
Tumor number			0.090	0.765
1	165 (72.7)	145 (74.0)		
≥ 2	62 (27.3)	51 (26.0)		
Tumor size (cm)			0.019	0.890
< 5	75 (33.0)	66 (33.7)		
≥ 5	152 (67.0)	130 (66.3)		
HTS grade			3.257	0.196
1	86 (37.9)	61 (31.1)		
2	81 (35.7)	86 (43.9)		
3	60 (26.4)	49 (25.0)		
PT (s)	12.30(11.50–13.10)	11.90(11.30–12.78)	1.569	0.117
INR	0.97(0.88–1.07)	1.00(0.93–1.06)	0.431	0.667
APTT (s)	33.00(28.90–37.70)	31.30(27.50–35.73)	1.281	0.201
White blood cell count (10^9/L)	6.34(5.01–8.27)	6.29(5.03–7.74)	1.080	0.281
Neutrophil count (10^9/L)	4.29(3.25–5.70)	4.40 (3.28–5.39)	0.829	0.408
Lymphocyte count (10^9/L)	1.52(1.10–1.88)	1.59(1.21–1.87)	-0.525	0.600
Platelet count (10^9/L)	199.00(161.00–259.00)	205.00(162.50–259.00)	-0.341	0.733
Hemoglobin count (g/L)	133.00(118.00–143.00)	132.20(121.00–144.00)	-1.379	0.169
Albumin (g/L)			4.509	0.034
≤ 30	211 (93.0)	191 (97.4)		
> 30	16 (7.0)	5 (2.6)		

*ICC* Intrahepatic cholangiocarcinoma, *ALT* Alanine Aminotransferase, *AST* Aspartate Aminotransferase, *AFP* Alpha fetoprotein, *CEA* Carcinoembryonic antigen, *CA19-9* Carbohydrate antigen 19–9, *PT* Prothrombin time, *APTT* Activated partial thromboplastin time, *INR* International normalized ratio

### Prognostic Implications of HALP, TBS, and HTS Grade

**Fig. 1:**
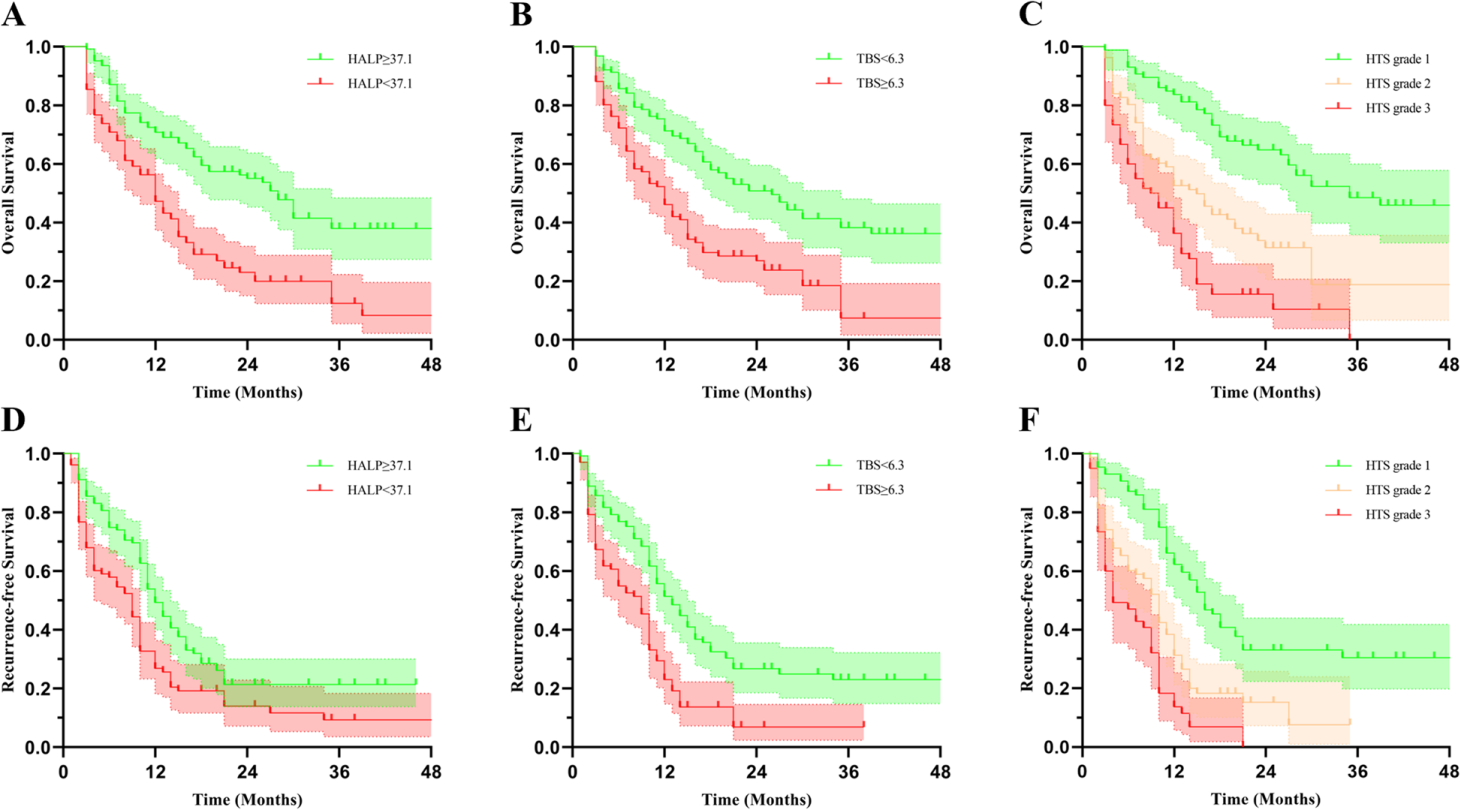
Kaplan–Meier curves for OS (**A**, **B**, **C**) and RFS (**D**, **E**, **F**) stratified in the training group by HALP, TBS, and HTS grade

**Fig. 2:**
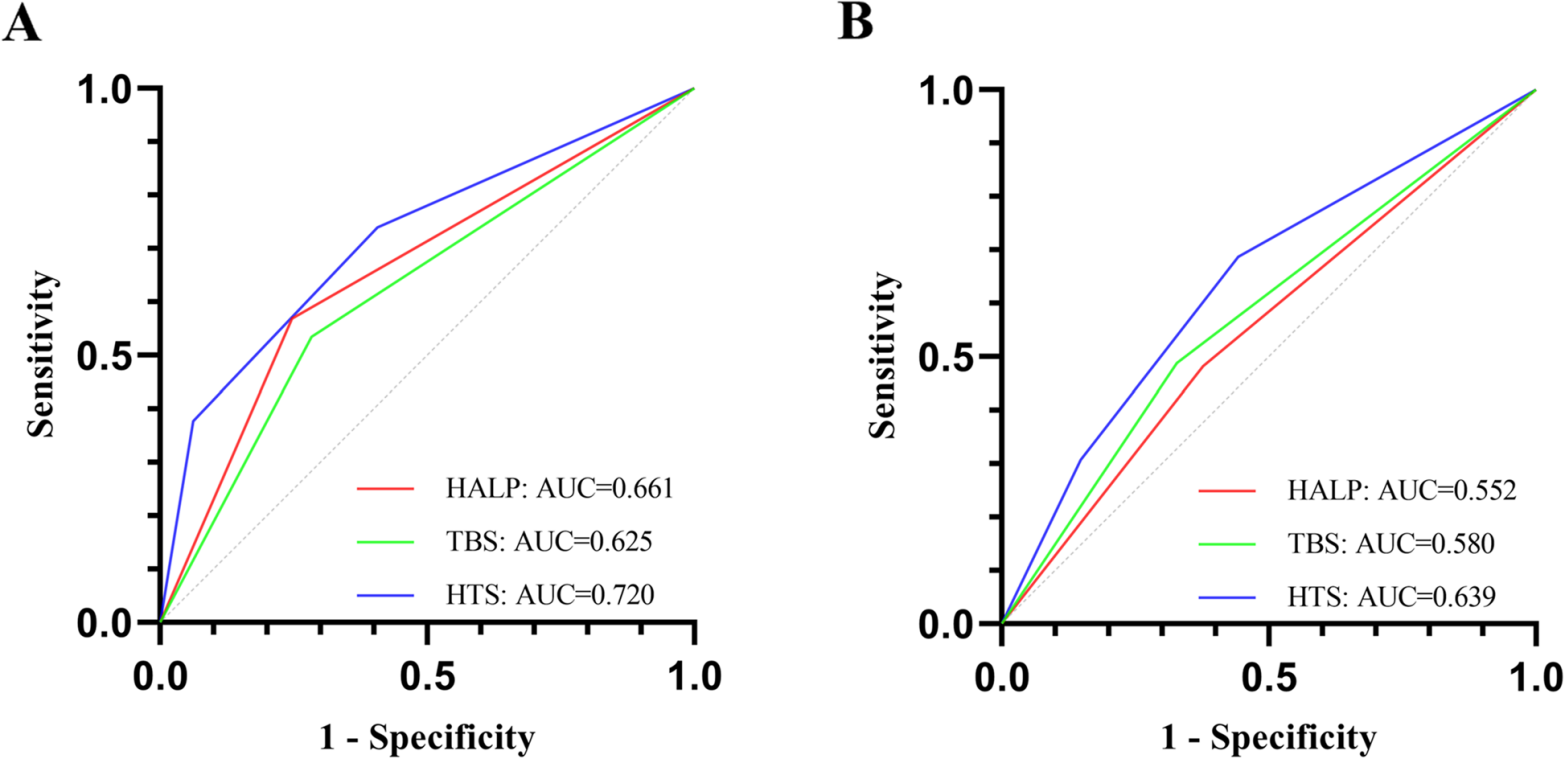
ROC curve comparison of predicted values using HALP, TBS, and HTS grade for OS (**A**) and RFS (**B**) in the training group

The cut-off values for HALP and TBS were 37.1 and 6.3, respectively. In univariate Cox regression analysis, history of viral hepatitis B, hepatolithiasis, Aspartate Aminotransferase (AST), bilirubin, Carcinoembryonic antigen (CEA), Carbohydrate antigen 19–9 (CA19-9), tumor differentiation, vascular invasion, perineural invasion, lymph node invasion, tumor number, tumor size, HALP, TBS, and HTS grade were each found to be influencing factors for OS (Table 3). History of viral hepatitis B, ALT, AST, CEA, CA19-9, tumor differentiation, tumor number, tumor size, vascular invasion, perineural invasion, lymph node invasion, HALP, TBS, and HTS grade were considered to be influencing factors for RFS (Table 4). In the training group, Kaplan–Meier curves showed that the prognosis was worse with low HALP and high TBS, and patients had increasingly worse OS and RFS as HTS grade increased (Fig. 1). Furthermore, our ROC results showed that HTS grade was more effective than HALP and TBS in predicting OS and RFS (Fig. 2). In the training group, the AUCs of HALP, TBS, and HTS for predicting OS were 0.661, 0.625, and 0.720, and HALP, TBS, and HTS predicted RFS with AUCs of 0.552, 0.580, and 0.639, respectively. Our multivariate results suggested that HTS grade, CA19-9, tumor differentiation, and vascular invasion were independent influencers of OS and that HTS grade, CA19-9, CEA, vascular invasion and lymph node invasion were independent influencers of RFS (Tables 3, 4).

**Table 3:** Cox regression analysis of OS in ICC patients in the training group

Factor	Univariate			Multivariate		
	HR	95%CI	*P*-Value	HR	95%CI	*P*-Value
Age, < 60/ ≥ 60	1.194	0.861–1.655	0.287			
Gender, male/female	0.852	0.614–1.182	0.338			
History of hepatitis B infection, -/ +	0.657	0.463–0.932	0.019	0.873	0.593–1.286	0.492
Hepatolithiasis, -/ +	1.697	1.110–2.596	0.015	1.261	0.780–2.037	0.344
ALT (U/L), < 50/ ≥ 50	1.412	0.981–2.034	0.064			
AST (U/L), < 40/ ≥ 40	1.748	1.242–2.461	0.001	0.889	0.562–1.405	0.613
Bilirubin (umol/L), < 21/ ≥ 21	1.782	1.190–2.667	0.005	1.615	0.971–2.689	0.065
AFP (ng/ml), < 7/ ≥ 7	0.849	0.603–1.196	0.349			
CEA (ng/ml), < 5/ ≥ 5	2.115	1.511–2.961	< 0.001	1.463	0.990–2.161	0.056
CA19-9 (U/ml), < 37/ ≥ 37	2.902	1.976–4.260	< 0.001	1.693	1.090–2.630	0.019
Tumor differentiation, well-moderate/poor	3.569	2.476–5.147	< 0.001	2.422	1.611–3.642	< 0.001
Tumor number, 1/ ≥ 2	1.809	1.273–2.571	0.001			
Tumor size (cm), < 5/ ≥ 5	1.735	1.201–2.508	0.003			
TBS ≥ 6.3	2.169	1.558–3.019	< 0.001			
PT (s)	1.131	1.006–1.271	0.052			
INR	0.980	0.890–1.079	0.685			
APTT (s)	0.997	0.969–1.025	0.819			
White blood cell count (10^9/L)	1.017	0.960–1.078	0.561			
Neutrophil count (10^9/L)	1.033	0.971–1.100	0.305			
HALP < 37.1	2.287	1.642–3.185	< 0.001			
Vascular invasion, -/ +	4.205	2.972–5.949	< 0.001	2.152	1.388–3.335	0.001
Perineural invasion, -/ +	2.212	1.586–3.086	< 0.001	1.433	0.984–2.087	0.061
Lymph node invasion, -/ +	2.297	1.564–3.374	< 0.001	1.331	0.863–2.053	0.196
HTS grade						
1						
2	2.480	1.615–3.807	< 0.001	1.626	1.023–2.583	0.040
3	4.639	3.026–7.111	< 0.001	2.462	1.503–4.032	< 0.001

*ICC* Intrahepatic cholangiocarcinoma, *OS* Overall survival, *ALT* Alanine Aminotransferase, *AST* Aspartate Aminotransferase, *AFP* Alpha fetoprotein, *CEA* Carcinoembryonic antigen, *CA19-9* Carbohydrate antigen 19–9, *PT* Prothrombin time, *APTT* Activated partial thromboplastin time, *INR* International normalized ratio, *HALP* Hemoglobin, albumin, lymphocyte, and platelet, *TBS* Tumor burden score, *HTS* HALP and TBS

**Table 4:** Cox regression analysis of RFS in ICC patients in the training group

Factor	Univariate			Multivariate		
	HR	95%CI	*P*-Value	HR	95%CI	*P*-Value
Age, < 60/ ≥ 60	1.076	0.791–1.463	0.640			
Gender, male/female	1.056	0.777–1.436	0.727			
History of hepatitis B infection, -/ +	0.647	0.466–0.897	0.009	1.071	0.749–1.532	0.707
Hepatolithiasis, -/ +	0.956	0.598–1.528	0.852			
ALT (U/L), < 50/ ≥ 50	1.536	1.086–2.172	0.015	1.282	0.788–2.087	0.317
AST (U/L), < 40/ ≥ 40	1.739	1.254–2.411	0.001	1.054	0.626–1.773	0.843
Bilirubin (umol/L), < 21/ ≥ 21	1.058	0.686–1.632	0.800			
AFP (ng/mL), < 7/ ≥ 7	1.012	0.738–1.387	0.941			
CEA (ng/mL), < 5/ ≥ 5	1.889	1.366–2.611	< 0.001	1.473	1.006–2.158	0.047
CA19-9(U/ml), < 37/ ≥ 37	2.480	1.770–3.476	< 0.001	1.580	1.066–2.341	0.023
Tumor differentiation, well-moderate/poor	1.790	1.240–2.583	0.002	1.368	0.915–2.047	0.127
Tumor number, 1/ ≥ 2	1.572	1.124–2.198	0.008			
Tumor size (cm), < 5/ ≥ 5	1.589	1.135–2.226	0.007			
TBS ≥ 6.3	1.992	1.460–2.718	< 0.001			
PT (s)	1.082	0.970–1.206	0.156			
INR	0.957	0.869–1.053	0.368			
APTT (s)	0.991	0.965–1.018	0.499			
White blood cell count (10^9/L)	1.024	0.971–1.081	0.381			
Neutrophil count (10^9/L)	1.037	0.978–1.099	0.222			
HALP < 37.1	1.646	1.212–2.237	0.001			
Vascular invasion, -/ +	2.801	2.033–3.858	< 0.001	1.804	1.211–2.689	0.004
Perineural invasion, -/ +	1.517	1.116–2.064	0.008	1.086	0.774–1.525	0.632
Lymph node invasion, -/ +	2.509	1.733–3.630	< 0.001	1.991	1.340–2.958	0.001
HTS grade						
1						
2	2.204	1.517–3.204	< 0.001	1.712	1.135–2.582	0.010
3	3.803	2.542–5.689	< 0.001	2.797	1.768–4.425	< 0.001

*ICC* Intrahepatic cholangiocarcinoma, *RFS* Recurrence-free survival, *ALT* Alanine Aminotransferase, *AST* Aspartate Aminotransferase, *AFP* Alpha fetoprotein, *CEA* Carcinoembryonic antigen, *CA19-9* Carbohydrate antigen 19–9, *PT* Prothrombin time, *APTT* Activated partial thromboplastin time, *INR* International normalized ratio, *HALP* Hemoglobin, albumin, lymphocyte, and platelet, *TBS* Tumor burden score, *HTS* HALP and TBS

### The Relationship between HTS grade and clinical information

Among all ICC patients, 147 (34.8%) were HTS grade 1, 167 (39.5%) were HTS grade 2, and 109 (25.8%) were HTS grade 3 (Table 5). Except for tumor size, tumor number, hemoglobin, albumin, lymphocyte, platelet, HALP, and TBS, HTS grades were significantly correlated with history of hepatitis B infection, high CEA, high CA19-9, Poorly differentiated tumor, vascular invasion, lymph node invasion, prothrombin time (PT) prolongation, elevated white blood cell and neutrophil count (all *P* < 0.05). Moreover, the clinicopathological features of patients with HTS high grade were the worst.

**Table 5:** The relationship between HTS classification and the clinical data of all ICC patients

Factors	HTS grade			*F*/*χ*^*2*^ Value	*P* Value
	1 (*n* = 147)	2 (*n* = 167)	3 (*n* = 109)		
Age ≥ 60 years	74 (50.3)	79 (47.3)	49 (45.0)	0.750	0.687
Male	85 (57.8)	90 (53.9)	59 (54.1)	0.573	0.751
History of hepatitis B infection	73 (49.7)	55 (32.9)	25 (22.9)	20.613	< 0.001
Hepatolithiasis	13 (8.8)	18 (10.8)	11 (10.1)	0.332	0.847
ALT ≥ 50 U/L	30 (20.4)	32 (19.2)	22 (20.2)	0.086	0.958
AST ≥ 40 U/L	29 (19.7)	47 (28.1)	33 (30.3)	4.454	0.108
Bilirubin ≥ 21 umol/L	19 (12.9)	27 (16.2)	21 (19.3)	1.910	0.385
AFP ≥ 7 ng/mL	45 (30.6)	54 (32.3)	27 (24.8)	1.878	0.391
CEA ≥ 5 ng/mL	42 (28.6)	53 (31.7)	53 (48.6)	12.347	0.002
CA19-9 ≥ 37 U/mL	73 (49.7)	105 (62.9)	93 (85.3)	34.745	< 0.001
Poorly differentiated tumor	26 (17.7)	47 (28.1)	36 (33.0)	8.514	0.014
Tumor number ≥ 2	18 (12.2)	52 (31.1)	43 (39.4)	26.419	< 0.001
Tumor size ≥ 5 cm	53 (36.1)	129 (77.2)	100 (91.7)	101.244	< 0.001
Vascular invasion	35 (23.8)	65 (38.9)	70 (64.2)	42.706	< 0.001
Perineural invasion	51 (34.7)	69 (41.3)	47 (43.1)	2.249	0.325
Lymph node invasion	35 (23.8)	69 (41.3)	39 (35.8)	10.965	0.004
HALP < 37.1	8 (5.4)	69 (41.3)	103 (94.5)	203.223	< 0.001
TBS ≥ 6.3	4 (2.7)	102 (61.1)	99 (90.8)	212.104	< 0.001
PT (s)	12.00(11.50–12.70)	11.90(11.10–13.00)	12.50(11.50–13.60)	3.695	0.026
INR	0.98(0.91–1.04)	0.97(0.89–1.04)	1.00(0.94–1.11)	0.790	0.454
APTT (s)	33.50(28.60–37.60)	31.60(27.90–35.60)	31.70(28.70–37.85)	0.923	0.398
White blood cell count (10^9/L)	5.80(4.87–7.26)	6.72(4.90–8.25)	6.56(5.63–8.30)	3.976	0.019
Neutrophil count (10^9/L)	3.59(2.82–4.50)	4.50(3.20–5.70)	4.63(3.91–6.46)	12.039	< 0.001
Lymphocyte count (10^9/L)	1.62(1.38–2.09)	1.62(1.17–1.98)	1.21(0.94–1.53)	30.448	< 0.001
Platelet count (10^9/L)	189.00(146.00–226.00)	206.00(161.00–259.00)	237.00(183.00–280.50)	15.262	< 0.001
Hemoglobin (g/L)	141.00(130.00–153.00)	130.00(116.00–138.00)	125.00(112.00–137.50)	27.947	< 0.001
Albumin (g/L)	42.40(38.70–45.60)	41.30(36.80–45.00)	39.20(35.85–43.40)	8.435	< 0.001

*ICC* Intrahepatic cholangiocarcinoma, *ALT* Alanine Aminotransferase, *AST* Aspartate Aminotransferase, *AFP* Alpha fetoprotein, *CEA* Carcinoembryonic antigen, *CA19-9* Carbohydrate antigen 19–9, *PT* Prothrombin time, *APTT* Activated partial thromboplastin time, *INR* International normalized ratio, *HALP* Hemoglobin, albumin, lymphocyte, and platelet, *TBS* Tumor burden score, *HTS* HALP and TBS

### Construction and validation of nomograms

**Fig. 3:**
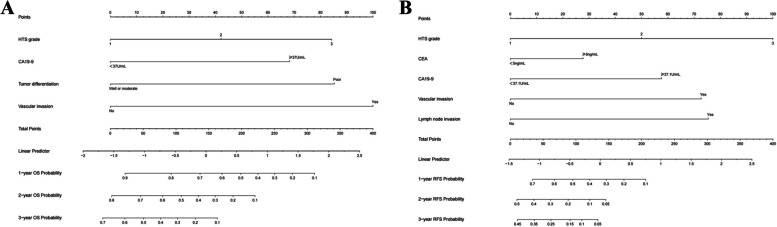
Prediction nomograms for OS (**A**) and RFS (**B**) in patients with ICC

**Fig. 4:**
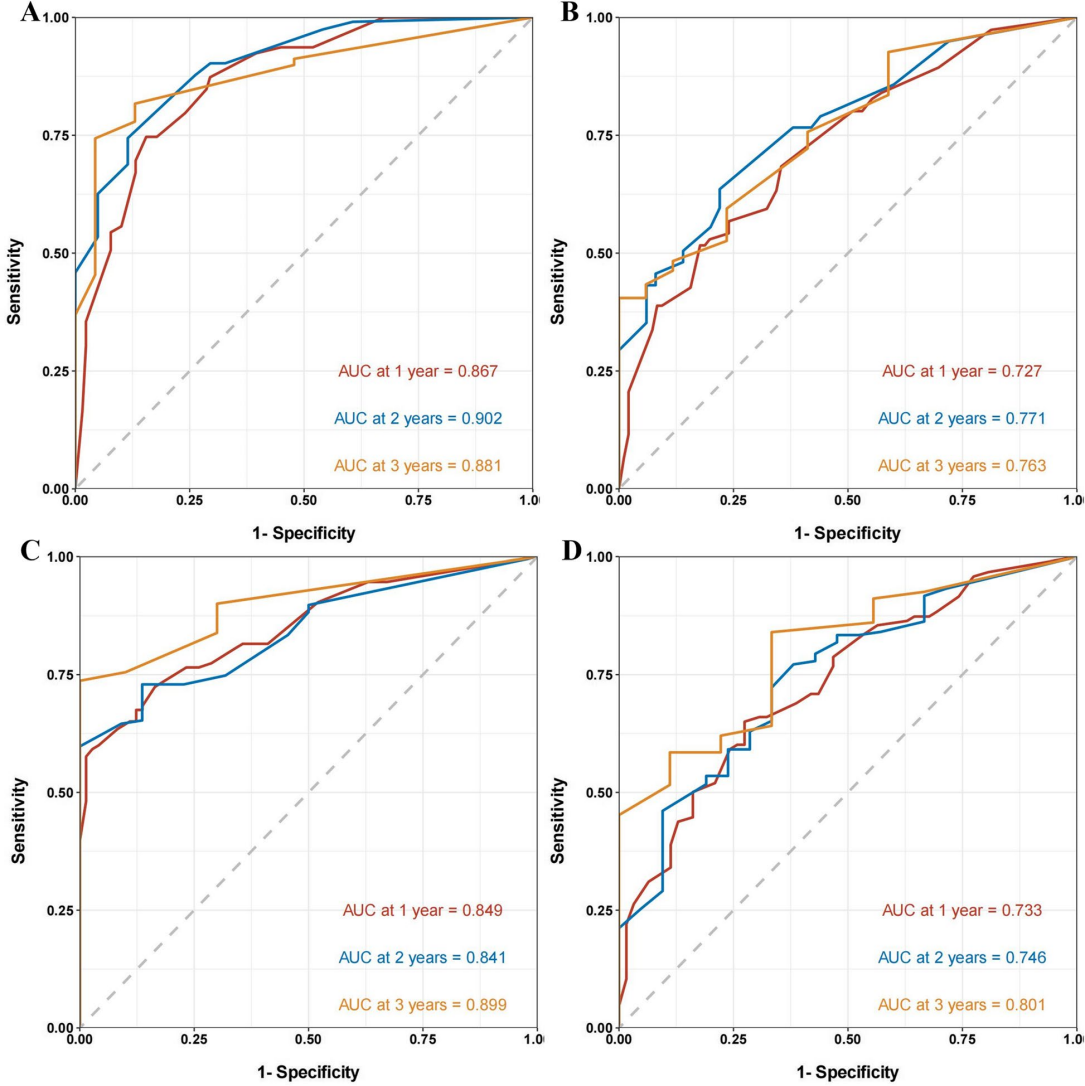
ROC curves of the nomograms. ROC curves for nomograms for OS in the training group (**A**) and validation group (**B**). ROC curves for nomograms for RFS in the training group (**C**) and validation group (**D**)

**Fig. 5:**
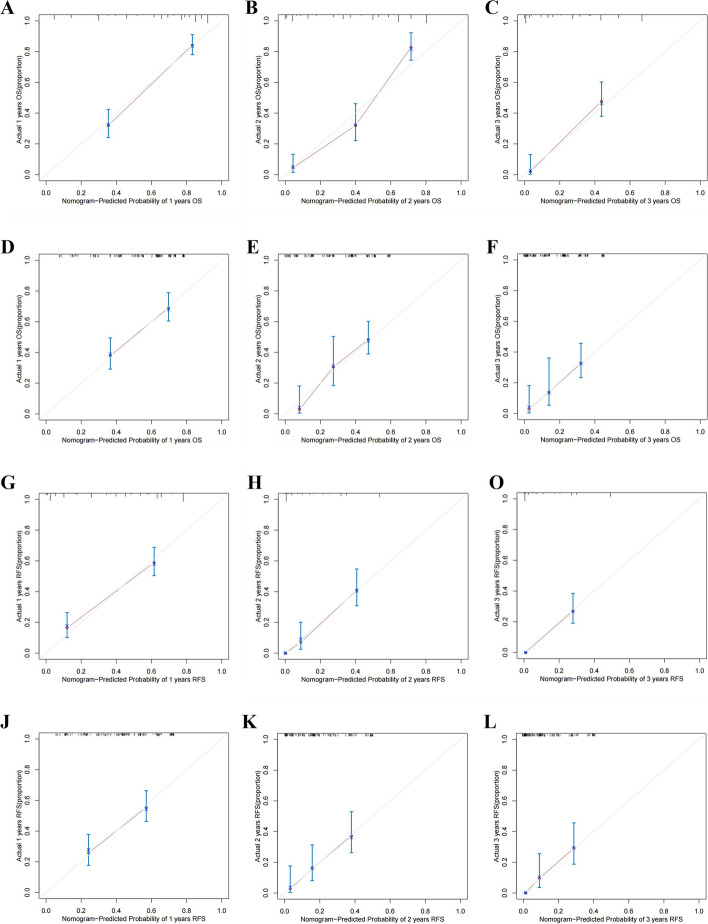
Calibration curves of nomograms in the third year. Calibration curves for nomograms for 1, 2 and 3 years OS in the training group (**A**,** B**,** C**) and validation group (**D**, **E**, **F**). Calibration curves for nomograms for 1, 2 and 3 years RFS in the training group (**G, H, I**) and validation group (**J, K, L**)

**Fig. 6:**
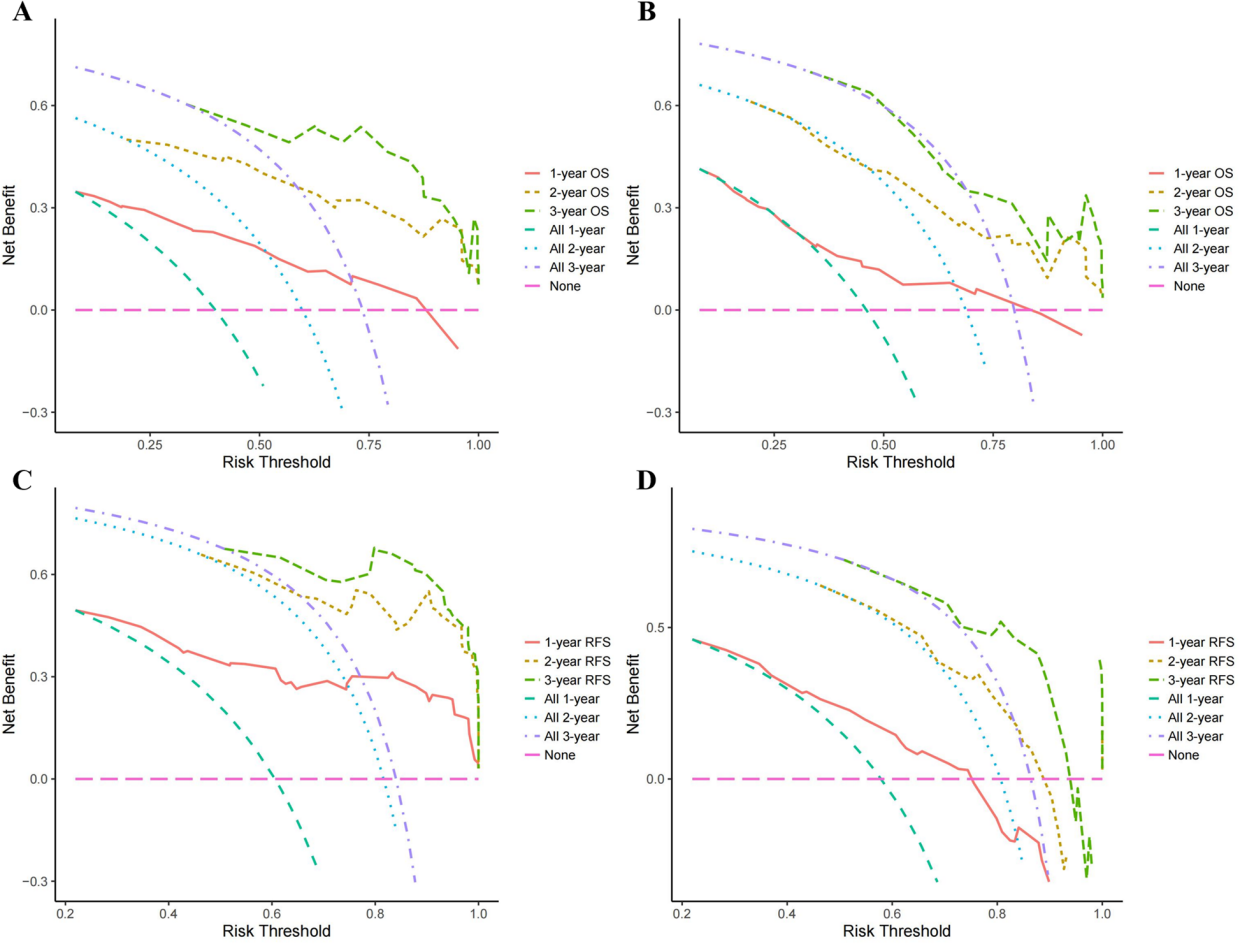
DCA of the nomograms. DCA for nomograms for OS in the training group (**A**) and validation group (**B**). DCA for nomograms for RFS in the training group (**C**) and validation group (**D**)

We next plotted the results of the multivariate Cox regression analysis into nomograms for predicted OS and RFS (Fig. 3). In the training group, the nomogram predicted that the AUCs of OS would be 0.867, 0.902, and 0.881 in the first, second, and third years after radical operation of ICC patients and that the AUCs of RFS would be 0.849, 0.841, and 0.899 in the first, second, and third years, respectively (Fig. 4A, C). In the validation group, the nomogram predicted that the AUCs of OS in the first, second, and third years would be 0.727, 0.771, and 0.763 respectively and that the AUCs of RFS in the first, second, and third years would be 0.733, 0.746, and 0.801 respectively (Fig. 4B, D), indicating that our models had excellent discriminant ability. Furthermore, the calibration curves of the OS and RFS nomograms in the training and validation group closely matched the 45° line, which indicated that the nomograms had a strong agreement between actual observations and predicted ones (Fig. 5). Finally, the results of our DCA showed that the nomograms had a significant positive net benefit, indicating that they should have important clinical value in predicting the OS and RFS of ICC (Fig. 6).

## Discussion

The aggressive nature of ICC creates a tremendous challenge for its diagnosis and treatment. In recent years, tremendous progress has been made in both of these areas, such as the improvement of imaging technology, immunotherapy and targeted therapy, but surgical resection remains the only effective treatment for ICC [19]. Radical resection that achieves negative margins can definitely improve the prognosis of patients [20]. However, due to the lack of specific clinical manifestations in the early stage of ICC, most patients reach the late stage of the disease when it is first discovered, and it is difficult to undergo radical surgical resection at this point [21]. Thus, such patients must seek other treatment methods, such as chemotherapy, targeted therapy, and immunotherapy. In this study, all the patients were confirmed by pathologists to have negative margins and to have achieved R0 resection. Therefore, set out to search for a preoperative index that can effectively predict the prognosis of ICC patients. To this end, we put forward a new index, HTS, which can simultaneously evaluate the inflammatory nutritional status and tumor morphology of patients. We constructed nomogram prediction models for ICC prognosis based on HTS grade and other independent influencing factors, and found that the HTS nomograms had excellent predictive value after validation.

Many studies have shown that the inflammatory response and nutritional status of the body are closely related to the prognosis of ICC tumors. For example, an increase in the ratio of neutrophil to lymphocyte count (NLR) is related to a lower OS for ICC patients [22]. The increase in systemic immune-infection index (SII) is also an independent risk factor for both OS and RFS in ICC patients [23]. Additionally, the prognostic nutritional index (PNI) and albumin-bilirubin (ALBI) are related to the occurrence of serious complications after ICC [24]. Thus, we introduced a new comprehensive index HALP that can reflect the inflammation and nutritional status of the body simultaneously. HALP can be obtained simply and cheaply as well because it is a common clinical indicator in hospitals.

Anemia is an independent prognostic factor for OS in ICC, and increasing hemoglobin levels can improve the oxygen saturation of a tumor, which can slow down its growth and thus improve the effectiveness of tumor treatment [25]. Hypoproteinemia and malnutrition result from the body's inflammatory response to malignancy; thus, serum albumin levels can reflect the severity of inflammation in tumor patients [26]. Additionally, lower levels of albumin are associated with reduced survival rates in malignancies [27]. Lymphocytes are important clinical indicators that reflect the immune status of patients and play an important role in the tumor immune response. They can mediate cytotoxic responses and release cytokines to inhibit tumor growth, proliferation, and metastasis [24]. Similarly, platelets can protect tumor cells from the elimination of the immune system and also promote tumor cells to extravasate, which may lead to the progress and metastasis of malignant tumors [28]. Previous studies have shown that HALP is superior to other inflammatory and nutritional markers, and HALP is also related to the occurrence of sarcopenia and to the immune microenvironment. Lower HALP levels are related to poor OS and RFS [11, 29], which is consistent with the research results in this study. However, another study has suggested that higher HALP means worse RFS, which is the opposite of our result [12]. This needs to be explored in depth by future studies.

In the 8th edition of the AJCC staging system, a tumor diameter of 5 cm is used as the boundary to divide the T1 category into T1a and T1b. The T2 category introduced tumor multiplicity and vascular invasion, demonstrating that tumor size and tumor number play a very important role in the prognosis of ICC [30]. TBS is a recently developed measurement method used to evaluate overall tumor burden that can combine the size and number of tumors based on the Pythagorean theorem. As an accurate method to assess overall tumor burden, TBS has subsequently been applied to several different cancers [31, 32]. In our univariate Cox analysis, tumor size, tumor number, and combined-index TBS were each found to be prognostic factors for ICC. Kaplan–Meier curves also showed that with elevated TBS, patients had worse OS and RFS, which is consistent with previous studies [15, 33]. Our study indicated that TBS derived from tumor size and number performed well in prognosis stratification of ICC patients after radical resection.

Both HALP and TBS were strong predictors of prognosis after radical resection. We also specifically studied the interaction between HALP and TBS and found that the HTS grade had better predictive performance than both of them. Moreover, we found that with the elevation of HTS grade, the proportion of patients with history of hepatitis B infection, high CEA, high CA19-9, positive vascular invasion, positive lymph node invasion and Other clinicopathological features increased. Patients with low HALP/ high TBS grade (HTS grade 3) had the worst OS and RFS, and patients with high HALP/ low TBS grade (HTS grade 1) had the best prognosis. HTS grade therefore has a positive value in the prognosis of ICC patients after radical resection.

Nomograms have unique advantages in prediction, such as their comprehensive and individual prediction ability, which allow for clinical guidance in personalized treatment of tumor patients [34, 35]. Simply put, clinicians calculate the corresponding scores by incorporating the patient's clinical data into a nomogram. They then determine the incidence of the corresponding outcome based on the total score obtained and make treatment mode decisions accordingly. Nomograms have been repeatedly found to be applicable to ICC and they had better predictive value than the traditional grading system [36, 37]. Therefore, we included HTS grade and other independent prognostic factors that were based on multivariate Cox regression analysis into our nomograms as well. We used ROC analysis, calibration curve analysis, and DCA to evaluate the predictive ability of the nomograms, and the results showed that they performed well in both the training and validation groups. For patients with ICC, if the nomograms predict a low risk of death or recurrence, they may opt for surgical treatment. On the other hand, if the risk is high, alternative treatment options such as chemotherapy or immunotherapy can be considered.

Undeniably, our research has some limitations. Because our study was retrospective, there may have been selection bias in choosing patients. The performance of our findings in a prospective study is also uncertain. Our study only included patients who underwent radical resection; therefore, the research results may not be applicable to all ICC patients. Cases that were unable to undergo radical resection or had negative margins were not included in the analysis, and this group of patients may have a poorer prognosis. In addition, although the multi-center nature of the study is an advantage, patient choice, the surgical techniques and medical resources of each participating center may have been different, which may have affected the results as well. Furthermore, all three medical institutions were located in China, and no evaluation of ethnic groups from other regions was conducted. Therefore, it is imperative to conduct prospective research involving multiple medical centers to further assess the predictive ability and accuracy of the nomogram based on HTS grade in determining the prognosis of ICC patients.

## Conclusion

In conclusion, this study demonstrated that HTS grade has significant predictive value for the prognosis of patients with resectable ICC, even more so than HALP and TBS, which showed synergistic effect in prognosis evaluation. The nomogram based on HTS classification is therefore a promising tool to stratify the prognosis of ICC patients after curative resection and may be able to help clinicians provide individualized clinical decision for ICC patients.

## Data Availability

The datasets analysed during the current study are available from the corresponding author on reasonable request.
